# Supplementary material for: Suppression of CCT3 Inhibits Tumor Progression by Impairing ATP Production and Cytoplasmic Translation in Lung Adenocarcinoma
Source: Int J Mol Sci. 2022 Apr 2;23(7):3983. doi: 10.3390/ijms23073983 (PMC9000022; doi:10.3390/ijms23073983)
Supplement: Supplementary file 1 [file ijms-23-03983-s001.zip › Supplementary figures.pdf]

Supplementary figures

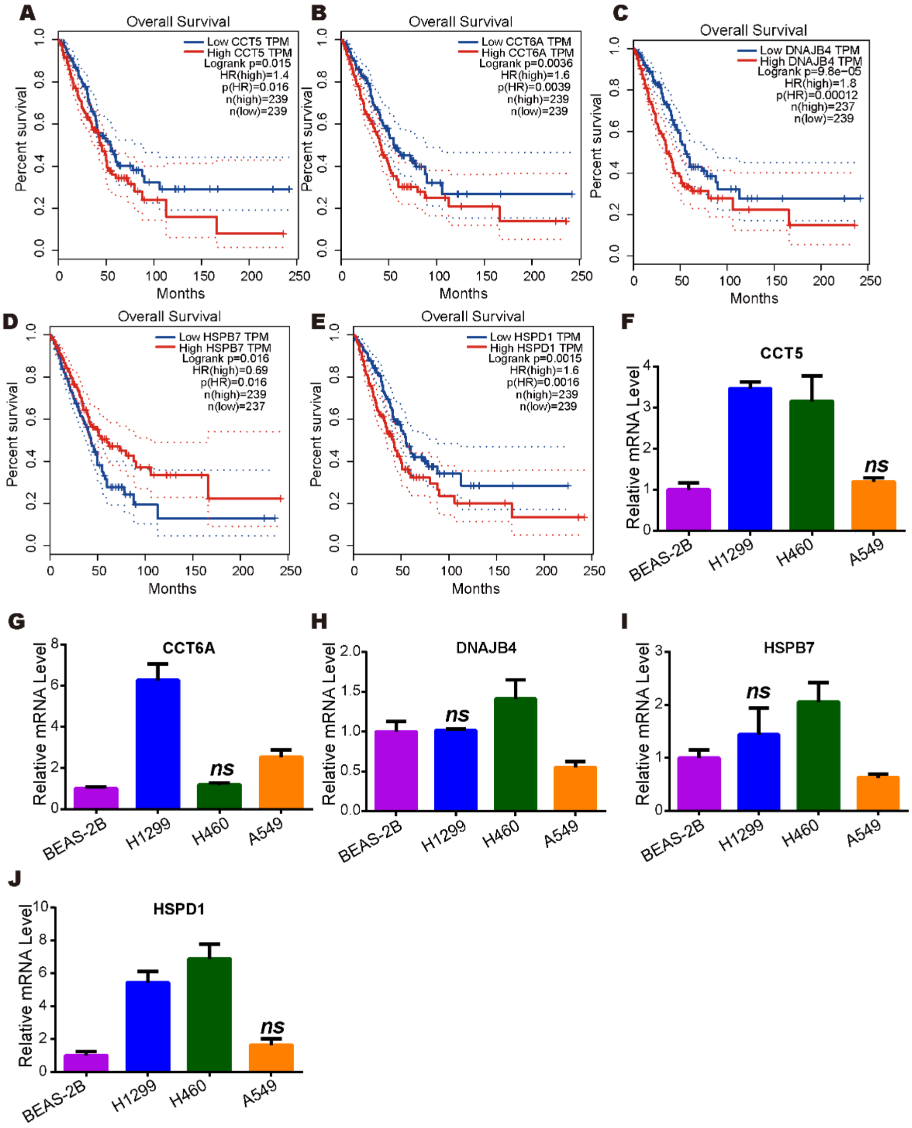

Supplementary Figure S1. The overall survival and relative mRNA levels of dysregulated HSP genes in LUAD. Related to Figure 1. (**A-E**) Kaplan-Meier curve for overall survival of LUAD patients with high or low expression of CCT5, CCT6A, DNAJB4, HSPB7 and HSPD1. HR, hazard ratio. The plots were performed by GEPIA. Differences were assessed using the log-rank test. The dashed lines represents the 95% confidence interval. (**F-J**) Relative mRNA levels of CCT5, CCT6A, DNAJB4, HSPB7 and HSPD1 in BEAS-2B, A549, H1299 and H460 cell lines. The values were normalized to the mRNA level of GAPDH. Representative data are from three independent experiments.

Data were shown as mean + SD, ns, not significant, * $p < 0.05$, ** $p < 0.01$, *** $p < 0.001$, **** $p < 0.0001$. Two-tailed Student's t-test.

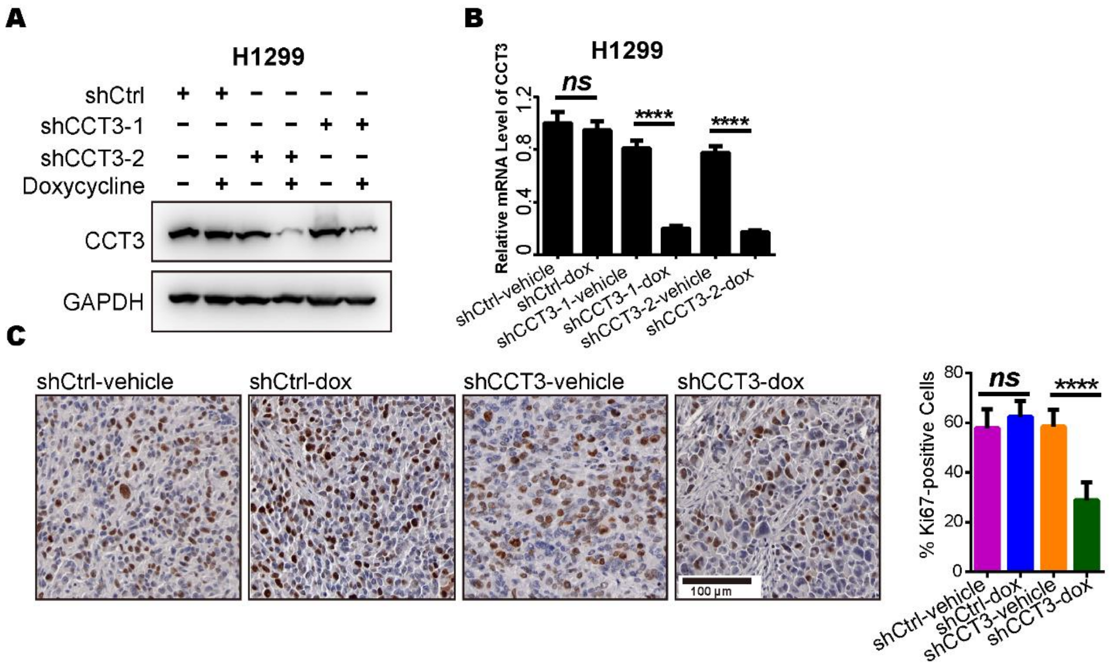

Supplementary Figure S2. The knockdown of CCT3 induced by doxycycline in H1299 cells. Related to Figure 2. (**A and B**) The efficiencies of CCT3 knockdown in H1299 cell lines harboring inducible shRNAs targeting *CCT3* or non-targeting control induced by doxycycline at protein (**A**) and mRNA (**B**) levels. The mRNA levels of CCT3 were normalized to the mRNA levels of GAPDH. Data were shown as mean + SD, ns, not significant, * $p < 0.05$, ** $p < 0.01$, *** $p < 0.001$, **** $p < 0.0001$.Two-tailed Student's *t*-test. (**C**) The percent of Ki67 positive cells in tumor tissues from subcutaneous tumor model (Figure **2A**). Representative images of IHC assays stained with anti-Ki67 antibody (left), quantification of Ki67 positive cells (right, n= 6 or 10/group). Scale bar = 100 μm. Data were shown as mean + SD, ns, not significant, **** $p < 0.0001$. Student's *t*-test.

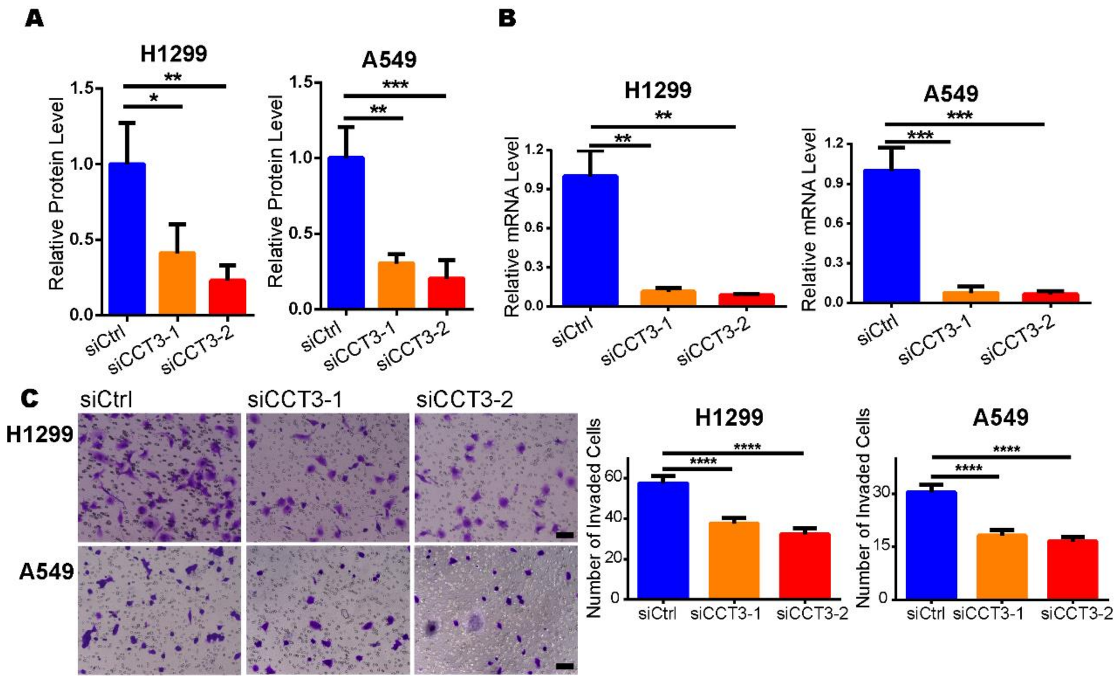

Supplementary Figure S3. Silencing CCT3 inhibits the invasion of LUAD cells. Related to Figure 3. (**A**) Quantification of protein levels (Figure 3A). Representative data are from three independent experiments. (**B**) Relative mRNA levels of CCT3 in H1299 and A549 cells treated with siRNAs targeting *CCT3* or a control. The values were normalized to the mRNA levels of GAPDH. (**C**) Representative images (left) and quantitative results (right) of the transwell assays to evaluate the invasion abilities of H1299 and A549 cell lines treated with siRNAs targeting *CCT3* or a control. At least 5 fields were quantitated in each well. Scale bar = 100 μm. Data are represented as mean + SD, * $p < 0.05$, ** $p < 0.01$, *** $p < 0.001$, **** $p < 0.0001$, two-tailed Student's *t*-test.

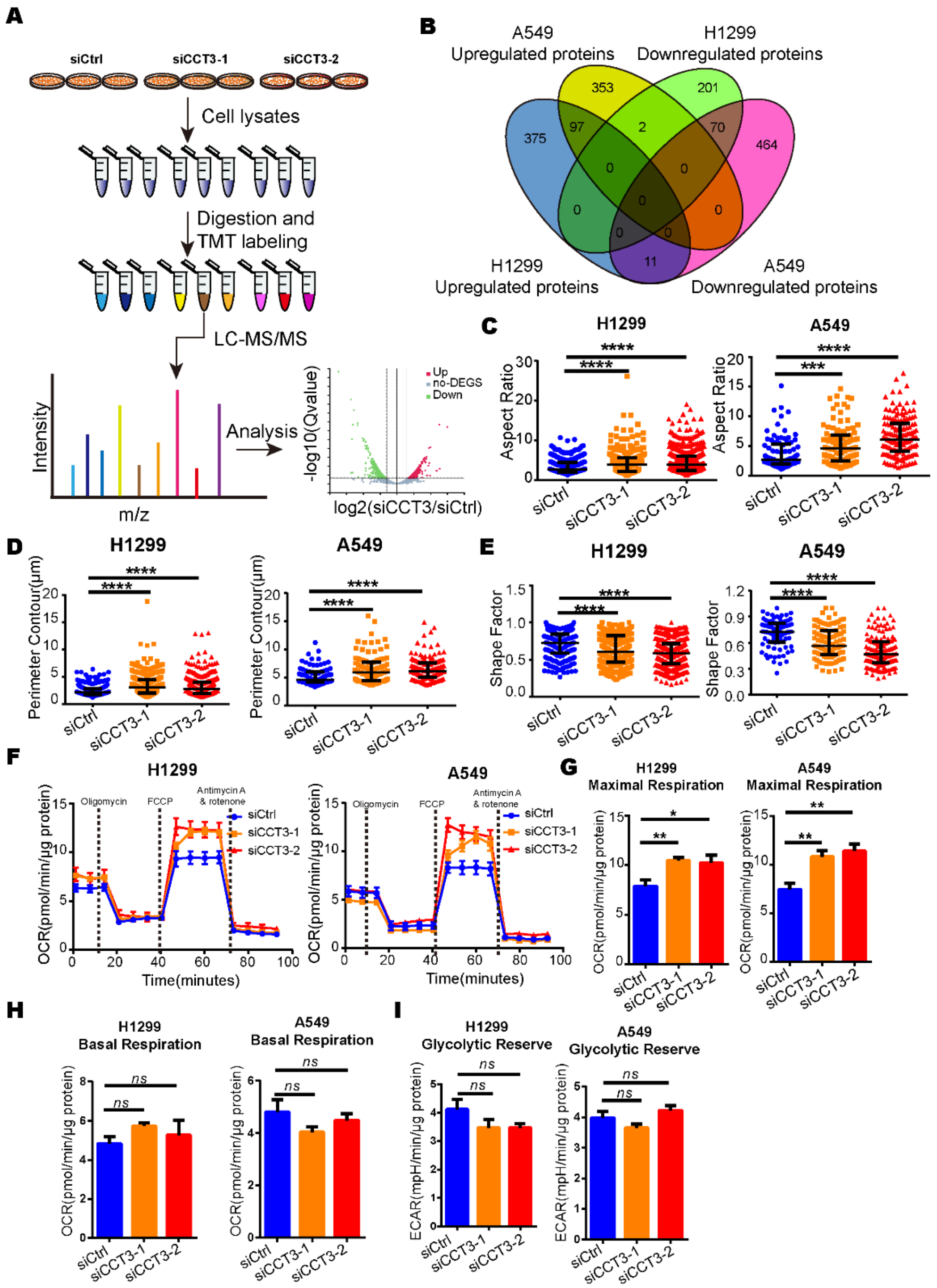

Supplementary Figure S4. Seahorse analysis of oxygen consumption rate and extracellular acidification rate in LUAD cells. Related to Figure 4. (**A**) The workflow of TMT-MS in H1299 and A549 cells between CCT3-deficient groups and control groups (n = 3/group). (**B**) Venn diagram of the dysregulated proteins in H1299 and A549 cells after CCT3 depletion. (**C-E**) Quantification of the aspect ratio (**C**), perimeter contour (**D**) and shape factor (**E**) of mitochondria in Figure 4B. Data were shown as median with interquartile range. *** $p < 0.001$, **** $p < 0.0001$. Mann-Whitney test. (**F**) Oxygen consumption rate (OCR) in indicated cells measured by Seahorse analysis (mitochondria stress test). Dashed lines represent the injection of corresponding components. Before experiment, the culture medium was replaced with XF assay medium supplemented with 10 mM glucose, 4 mM glutamine and 2 mM pyruvate, The final concentrations of compounds were: 4 μM oligomycin, 1 μM carbonyl cyanide 4-

(trifluoromethoxy)phenylhydrazone (FCCP) and 1 μM antimycin A/rotenone. Data are represented as mean ± SEM. (**G-H**) Functions in maximal respiration (**G**) and basal respiration (**H**) of mitochondria evaluated by OCR in CCT3 knockdown and control cells from (F). Data was normalized to protein quantity and represented as mean + SEM, * $p < 0.05$, ** $p < 0.01$, two-tailed Student's *t*-test. (**I**) Function of glycolytic reserve evaluated by ECAR in CCT3 knockdown and control cells. The values were normalized to protein quantity quantified by glycolysis stress assay. Data were normalized to protein quantity and represented as mean + SEM, ns, not significant, two-tailed Student's *t*-test.

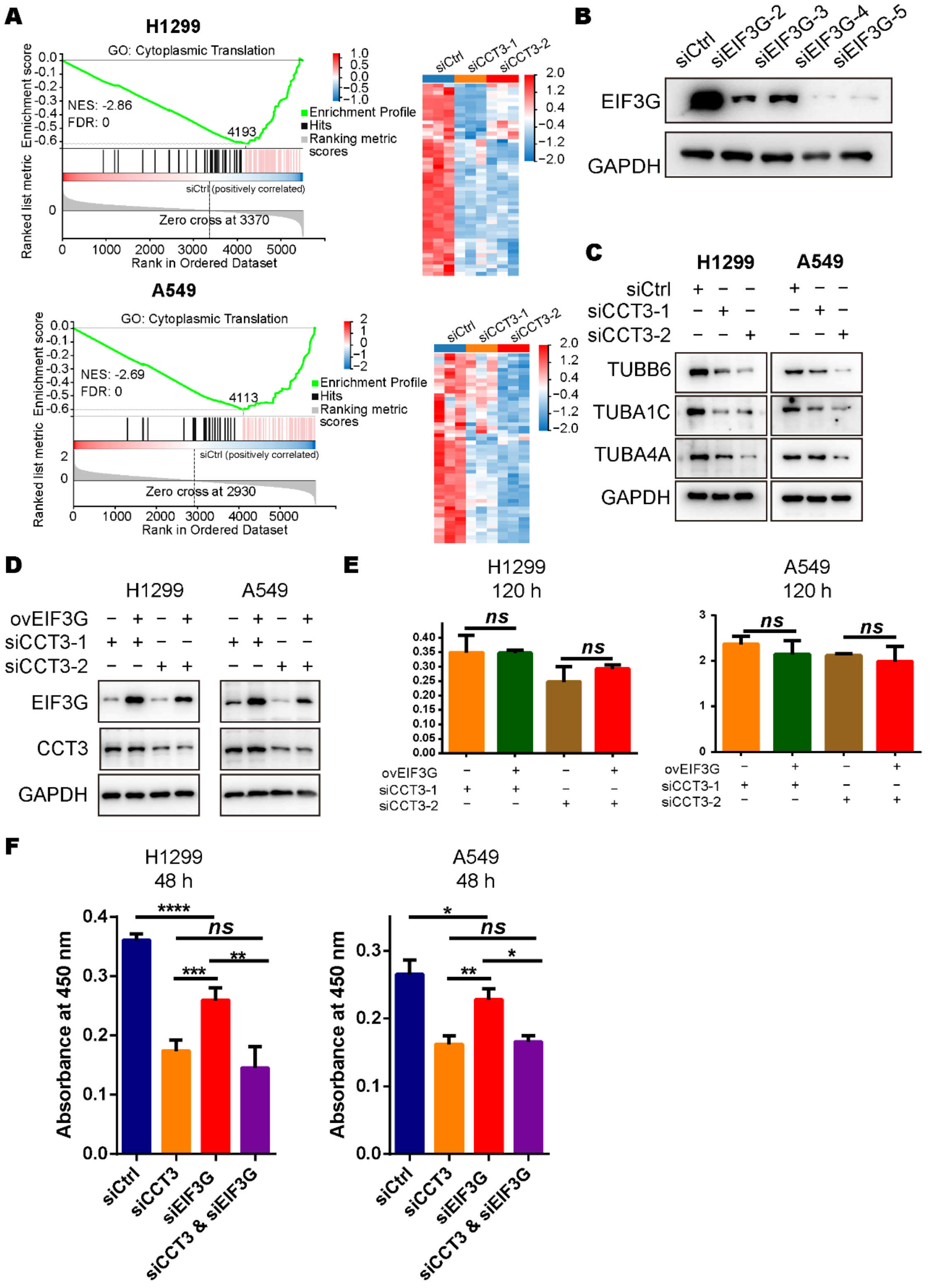

Supplementary Figure S5 Suppression of CCT3 down-regulated the proteins involved in cytoplasmic translation pathway in LUAD cells. Related to Figure 5 and 6. (**A**) Enrichment plots (left) of the cytoplasmic translation pathway from GSEA, and heat maps (right) of the corresponding down-regulated proteins in CCT3 knockdown groups. The red color represents overexpression and the blue symbolizes downregulation. (**B**) The efficiencies of EIF3G knockdown with four siRNAs targeting *EIF3G* in A549 cells.

(**C**) Representative WB images of TUBB6, TUBA1C and TUBA4A after CCT3 knockdown. (**D**) Representative WB images of EIF3G overexpression in CCT3-deficient LUAD cells. (**E** and **F**) Cell proliferation in indicated treatments measured with CCK8 assays. Data were shown as mean + SD, ns, not significant, * $p < 0.05$, ** $p < 0.01$, *** $p < 0.001$, **** $p < 0.0001$, two-tailed Student's t-test.
